# Cantilever-enhanced photoacoustic measurement of HTO in water vapor

**DOI:** 10.1016/j.pacs.2022.100443

**Published:** 2022-12-30

**Authors:** Juho Karhu, Markus Nyman, Marja Siitari-Kauppi, Tuomas Hieta

**Affiliations:** a Metrology Research Institute, Aalto University, Maarintie 8, Espoo, 02150, Finland; b Department of Chemistry, University of Helsinki, P.O. Box 55, Helsinki, 00014, Finland; c Gasera Ltd., Lemminkäisenkatu 59, Turku, 20520, Finland

**Keywords:** Cantilever-enhanced photoacoustics, Gas sensing, Tritiated water

## Abstract

A photoacoustic detection of tritiated water (HTO) is presented. The method uses cantilever-enhanced photoacoustic spectroscopy (CEPAS) to reach sub-ppb sensitivity for HTO in the gas phase. A noise equivalent concentration of 0.88 ppb is reached with a sampling time of 1 min. The high performance and small sample volume of CEPAS allows sensitive detection of HTO from a sample with low total activity.

## 1 Introduction

There is increasing interest in studying the application of isotopologue specific high-resolution spectroscopy in gas-phase measurement of radioactive compounds. Previously, measurements of carbon-14 have been of particular interest, since it is one of the common emissions from nuclear sites and ^14^C measurements have applications in carbon dating and emission source apportioning. Various spectroscopic detection methods for radiocarbon compounds have been demonstrated in the recent past [1], [2], [3], [4], [5], [6], [7], with the some techniques starting to reach the detection limits well below the natural abundance of ^14^C, which is required in many applications [8]. Methods based on laser spectroscopy offer the opportunity of developing detectors capable of performing sensitive and continuous on-site monitoring of radioactive compounds at nuclear facilities with fast time response [9].

Tritium (^3^H or T) is another highly important radioisotope, which is present in nuclear fission and fusion environments and a major contributor to the total radioactive release in gaseous and liquid emissions from nuclear facilities [10]. There has been several reports for development of spectroscopic detectors for tritiated water (HTO), based on wavelength modulation spectroscopy [11], [12] and cavity ring-down spectroscopy (CRDS) [13]. A noise equivalent detection limit of 4 ppb was demonstrated using multipass wavelength modulation spectroscopy at the wavelength 7.9 µm with a measurement time of a few minutes^1^ and 66 ppb using a CRDS measurement at the wavelength 2.2 µm with a measurement time of 1 min [11], [13].

Spectroscopic data on HTO is sparse, but several FTIR studies have started to assign HTO absorption bands [14], [15], [16], [17] and an extensive line list based on theoretical calculations [18], [19] is accessible through the SPECTRA database [20]. In this article, we present a highly sensitive method for gaseous HTO detection based on cantilever-enhanced photoacoustic spectroscopy (CEPAS) [21]. In CEPAS, the photoacoustic signal is recorded with a cantilever microphone, which bends in response to the acoustic signal, instead of stretching like condenser microphones. This typically provides over an order of magnitude improvement in sensitivity compared to conventional microphones. Optical techniques often require relatively large sample volumes, because the sensitivity is proportional to the absorption path length, but the CEPAS technique is particularly suitable when working with radioactive samples, because it offers high sensitivity in a small sample volume, so that the total radioactivity of the sample can be kept low [7]. We perform spectral measurements to identify a HTO absorption line suitable for spectroscopic detection, as well as demonstrate the practical detection capability of the setup.

## 2 Experimental methods

**Fig. 1:**
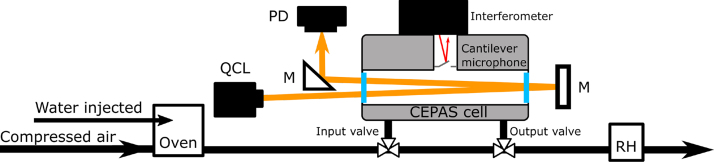
Schematic picture of the measurement setup. The light source is a continuous-wave QCL and the optical power is monitored with a thermopile power detector (PD). To increase the optical power inside the photoacoustic cell, the laser beam is reflected a second time through the cell with gold mirrors (M). The photoacoustic signal is detected with a cantilever-microphone. The movement of the microphone is measured optically with an interferometer. The water sample is generated by mixing compressed air with water vapor from an evaporator, which consists of a step-motor controlled syringe feeding water into a 140 °C oven. The water vapor concentration in the sample flow is measured with a humidity sensor (RH).

A schematic picture of the measurement setup is presented in Fig. 1. The detection system is based on a commercial photoacoustic cell (PA201, Gasera) and the setup is similar to that used previously for trace gas measurements, for example in [22]. The photoacoustic cell is 8 cm long and 4 mm in diameter. The cell is heated up to a temperature of 50 °C to ensure that water will not condense inside the cell. The cantilever microphone is located at the midpoint of the cell. The movement of the cantilever is recorded with an interferometer [23]. The light source is a continuous-wave quantum cascade laser (QCL) emitting at the wavelength 7.32 µm (AdTech Optics). After passing through the photoacoustic cell, the laser is reflected back through the cell a second time, to increase the total optical power. The power is recorded with a thermopile detector (greenTEG). The total optical power inside the photoacoustic cell, when accounting for the double-pass configuration, is 125 mW. The CEPAS measurement is performed in sample-and-hold configuration. That is, a sample is transferred into the CEPAS cell from a continuous by-pass flow, and the cell valves are then closed for the duration of the spectral measurement. The pressure inside the cell during the spectroscopic measurement is 200 mbar to decrease the pressure linewidths and reduce interference from neighboring lines.

The gaseous samples are generated by mixing compressed air with water vapor from a custom evaporator. The custom evaporator consists of a step-motor syringe pump and an oven. The liquid samples are rapidly evaporated in the oven at 140 °C and mixed with compressed air flow that acts as the carrier. A water sample with enriched amount of HTO is used for the HTO measurements. The tritiated water was purchased from Perkin Elmer (Boston, USA). The activity concentration of the HTO water sample is reported as 37 MBq g^−1^ (1.4.2022), with a 10 % uncertainty. The sample volume available to us was limited due to radiation safety concerns. The activity corresponds to a relative mole fraction of 6.05 × 10^-7^ mol/mol for the HTO concentration to the total water concentration in the gas phase when the sample is evaporated. Ion-exchanged water was used as a background reference, to distinguish the spectrum of HTO from the stable water isotopologues. The total water concentration in the sample flow is monitored with a humidity sensor (ChipCap 2, Telaire).

**Fig. 2:**
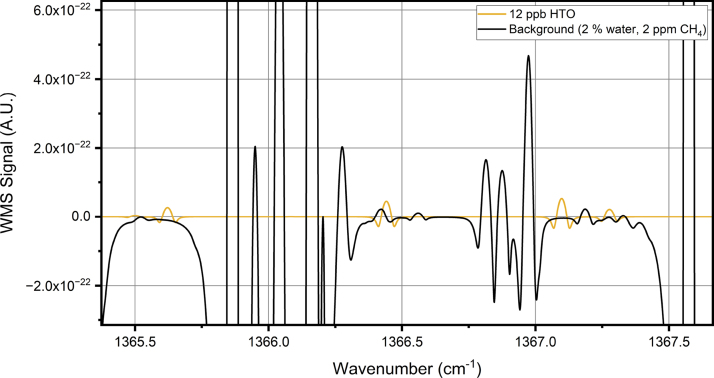
Simulation of a wavelength modulation spectrum [24] of 12 ppb of HTO and expected background, which consists of 2 % of water and 2 ppm of methane. The HTO line parameters are based on the simulated line list from the SPECTRA database and the other line parameters are from the HITRAN database. The pressure was set to 200 mbar and the modulation amplitude to 0.03 cm^−1^ for the simulation.

The photoacoustic spectrum is measured with wavelength modulation [24], and the signal is detected at the second harmonic of the modulation frequency. Injection current is used to scan and modulate the wavelength of the QCL. The modulation frequency was 80 Hz. The signal-to-noise ratio of the cantilever microphone is relatively independent of the modulation frequency, up to the resonance frequency of the cantilever at approximately 700 Hz, and here the detection frequency is chosen based on where environmental acoustic noise is low. We first performed spectral scans to identify HTO absorption lines. A spectrum of water with normal isotope distribution is first measured from ion-exchanged water, to generate a background spectrum of the stable isotopologues. This spectrum is also used to calibrate the wavelength axis of the spectra, based on the known water line positions reported in HITRAN [25]. Another spectral measurement with the HTO enriched water is performed, and by subtracting the background spectrum measured with stable isotopologues, a spectrum of only HTO is obtained. Four potential lines of HTO with low interference from stable water isotopologues were identified within the tuning range of the laser according to simulations of the theoretical linelist of HTO, together with HITRAN data (Fig. 2). We performed spectral scans over the predicted positions of the HTO lines at 1365.621 cm^−1^, 1366.441 cm^−1^ and 1367.098 cm^−1^, but due to the low concentration of HTO in our sample, we were able to properly identify only the line predicted to be at 1366.441 cm^−1^. In the simulation, the line at 1367.098 cm^−1^ appeared potentially better, but we detected it at slightly lower wavenumber with a very low signal-to-noise ratio, mostly due to interference from the much stronger water line next to it.

After verifying the HTO spectral response, time-series measurements with varying water and HTO concentrations is carried out to evaluate the practical performance of the detection scheme. A background spectrum of the stable water isotopologues and a HTO spectrum with a known HTO concentration are used as library spectra for HTO detection. The background library spectrum is again generated with an ion-exchanged water sample. A library spectrum of HTO is taken as an average of three spectral scans with a known HTO concentration, after subtracting the background spectrum. The concentration of HTO in the library spectrum is calculated according to the known activity of the HTO sample, and the measured water vapor concentration. All other measured spectra in the time series are fitted with the two library spectra using classical least squares (CLS) to calculate the HTO concentration over time.

## 3 Results and discussion

**Fig. 3:**
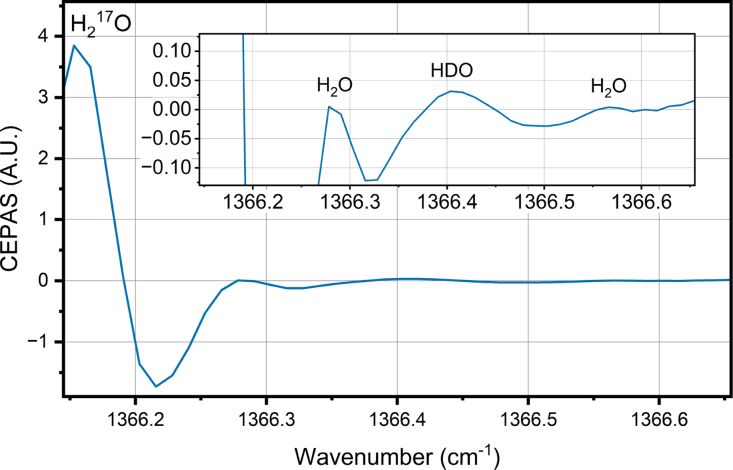
Wavelength modulation spectrum of the measurement background. The background consists mostly of water with normal isotope distribution, which is added to the sample flow with an evaporator. The water concentration is 2 %. There are also some features arising from other trace gases present in the compressed air, which is used as the carrier gas.

**Fig. 4:**
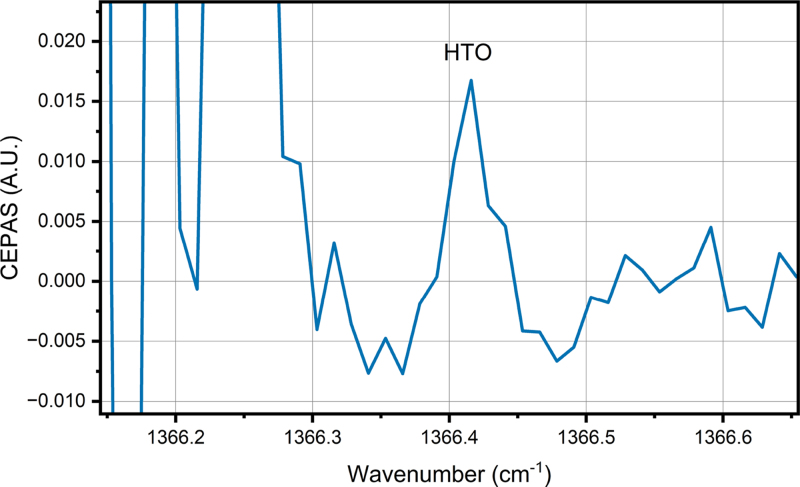
Wavelength modulation spectrum of the HTO. The spectrum is measured from a sample flow where HTO enriched water has been added with an evaporator. The total water concentration is 2 %, and relative HTO concentration of the total water vapor is 6.05 × 10^-7^ mol/mol. The background spectrum presented in Fig. 3 has been subtracted to get rid of the stable water isotopologues. The noise below 1366.2 cm^−1^ is due to the strong H_2_^17^O line: because of the very strong signal, small uncertainties in the signal level and laser wavelength produce high noise in the difference spectrum.

Fig. 3 shows the measured water background spectrum. Fig. 4 shows the HTO spectrum, where the contribution of the stable water isotopologues has been subtracted out, showing only the target HTO line, which is detected at line position 1366.416 cm^−1^. The background spectrum is first scaled according to the height of the strong line at 1366.16 cm^−1^ as the water concentration between the two measurements are slightly different.

The HTO line overlaps with another weak water line belonging to HDO isotopologue. To verify that the detected HTO in the difference spectrum is not simply due to a difference in relative abundance of HDO between our ion-exchanged water sample and the HTO sample, we verified that the height of the much stronger HDO line at 1367.574 cm^−1^ was the same for both samples, to within 3 %, when total water concentration measured with the humidity sensor was the same. It should be noted that the height of the HTO line is approximately half of the HDO line, and such a difference would be clearly visible for the stronger HDO line. We also verified that the same was true for the line at 1366.16 cm^−1^, because it belongs to the H_2_^17^O isotopologue. If the H_2_^17^O abundance was different between the two samples, scaling the background spectrum according to that line could lead to incorrect subtraction of other stable water isotopologues. The position of the line detected in the difference spectrum was close to the predicted HTO line position, with a difference of 0.025 cm^−1^, which is in line with differences observed in a previous study [13]. The height of the line, relative to the lines of the stable water isotopologues, is smaller than predicted based on the simulation in Fig. 2. The cause for this could be the uncertainty of the line strength in the theoretical line list. Another possibility is that part of the sample activity is due to T_2_O, since our tritiated water sample did not differentiate between the tritium isotopologues. Our simulation, and the following calculations of HTO concentration, assume that the tritium is all in HTO form, which may have led to an underestimation of the detection performance.

**Fig. 5:**
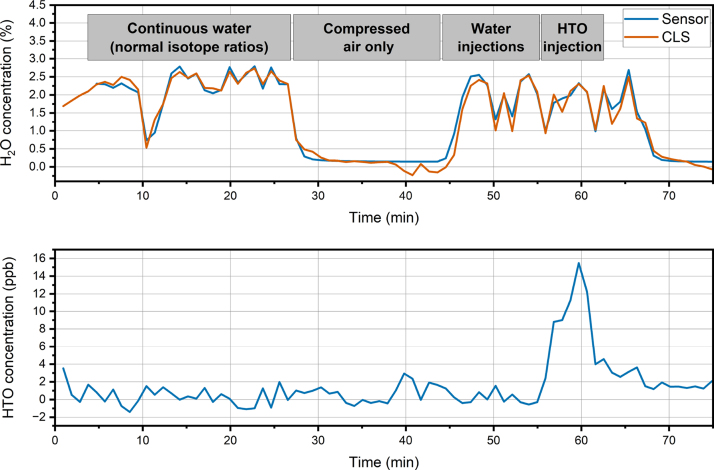
A HTO concentration measurement time series, showing the water concentration (above) and the HTO concentration measured with CEPAS (below). The water concentration is shown as measured with both a humidity sensor (Sensor) and from the CLS fit (CLS), which agree well. The water and HTO concentration of the sample flow was varied to evaluate the practical performance of the method (see text for details).

In time series concentration measurements, the sample inside the CEPAS cell is renewed every 60 s. The laser wavelength is scanned over the strong water line at 1366.16 cm^−1^ and the HTO line, and a spectrum of 20 points in total is measured from each sample over 30 s. The rest of the time is spent on the gas exchange, laser tuning and stabilization. The wavelength axis of each scan is referenced to the well-known position of the water line at 1366.16 cm^−1^, to counteract possible drifts in the laser wavelength over time. Fig. 5 shows a 70 min measurement set performed with the setup. Compressed air was continuously flowing though the oven and pass the CEPAS sample input valve with a rate of 1 l min^−1^. The HTO concentration was continuously calculated from the CLS fit. The CLS fit also gives the total water concentration through the fit of the water background spectrum. Fig. 5 shows that the water concentration from the humidity sensor agree well with the water concentration given by the CLS fit. This shows that the background compensation is working properly, which eliminates the interference from the HDO line overlapping with the HTO line during the time series measurement. For 20 min in the beginning of the set, water with normal isotope ratio was injected into the carrier gas flow (*continuous water* phase in Fig. 5). The water concentration was measured to be approximately 2.5 % in volume mixing ratio. The water background spectrum used in the CLS fit was taken from this part of the time series. The measured HTO concentration remained at zero as expected. For the next 20 min, the water injection was stopped altogether, so that the sample was dry compressed air (*compressed air only* phase in Fig. 5). It should be noted that the compressed air used as the carrier gas had 0.15 % of water vapor, as measured by the humidity sensor. Water was then again injected into the oven for 10 min (*water injections* phase in Fig. 5). The water concentration in the flow is less stable now, because a smaller volume syringe, which is needed for injection of the low-volume HTO enriched sample, was used and had to be refilled once during the 10 min period. Despite the larger fluctuations, the good agreement between the humidity sensors and the water concentration from the CLS shows that the water concentration is still measured properly. Next, a HTO enriched water sample was injected into the flow for 5 min (*HTO injections* phase in Fig. 5). A clear increase in the detected HTO concentration is observed. The library spectrum for HTO used in the CLS fit was taken from the average of the three samples, where the highest HTO concentrations were recorded around the 60 min mark. The average water concentration was measured to be approximately 2.32 % during those time steps. However, we know that 0.15 % was due to the humidity of the compressed air, so the total water concentration from the sample generation was only 2.17 %. The fraction of HTO concentration to the total water concentration is 6.05 × 10^-7^, which gives us a HTO concentration of 13 ppb in volume mixing ratio. After the HTO injection, the system was flushed with a final injection of water with normal isotope ratios.

**Fig. 6:**
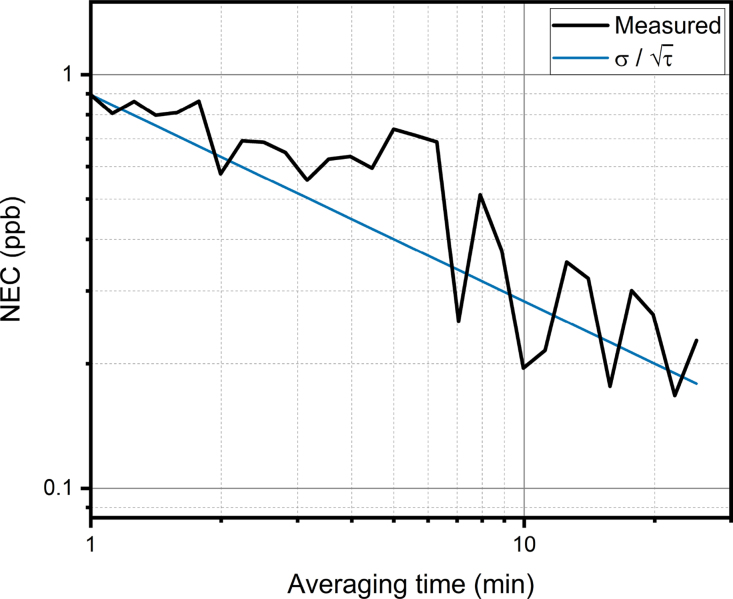
Allan-Werle deviation of the background measurement. The black line shows the measured noise equivalent concentration as a function of the averaging time and the blue line shows the theoretical white noise averaging.

Due to the limited amount and low activity of our sample, we are not able to perform a complete analysis of the sensitivity and linearity with a set of different HTO concentrations, but we can evaluate the performance and detection limit based on the signal-to-noise ratio of the measurement. The signal-to-noise ratio, as calculated from the measured HTO concentration and the standard deviation of the measurements before the HTO injection, was approximately 15, which results in a noise equivalent concentration is then 0.88 ppb. Due to the limited amount of HTO sample available to us, we were not able to evaluate the stability of the measurement with a HTO sample. Fig. 6 shows the Allan-Werle deviation [26] calculated from the background measurements before the HTO injection. The figure shows that the measurement is relatively stable throughout the background measurements. It should be noted that the data used for the stability evaluation includes the *continuous water*, *compressed air* and *water injections* phases as described in Fig. 5. Even substantial changes in the water concentration did not cause significant changes in the HTO response.

**Table 1:** Measurement conditions and detection performance parameters of the setup. The noise equivalent concentration of the setup is also shown as the total number of HTO molecules inside the measurement cell and the corresponding activity.

Parameter	Value
Cell volume	11 ml
Sample pressure	200 mbar
Cell temperature	323 K
Noise equivalent concentration	0.88 ppb
HTO molecules in the cell	4.3 × 10^10^
Corresponding activity	78 Bq

## 4 Conclusions

We have demonstrated an optical HTO detector based on cantilever-enhanced photoacoustic spectroscopy. The noise equivalent detection limit is 0.88 ppb of HTO in air, with a 60 s sample time, which includes also the time spent on the gas exchange between the spectroscopic measurements. This is, to our knowledge, the best sensitivity demonstrated for HTO detection with optical methods. A concentration of 0.88 ppb of HTO corresponds to an absorption of 6.35 × 10^-9^ cm^−1^ according to simulations with the data available from the SPECTRA database. The optical power was 0.125 W and the measurement time 60 s, which gives a normalized noise equivalent absorption (NNEA) of 6.15 × 10^-9^ cm-1 WHz-1/2. This is somewhat high compared to typical NNEA achieved with CEPAS, but it should be noted that the result is affected by the uncertainty in the simulated line intensity and in the HTO concentration calculated from the total activity. We have also used the total measurement time in the NNEA calculation, instead of only the time used for the spectral scan, for more fair comparison to other methods.

The total sample volume of the CEPAS cell is only approximately 11 ml, so the method is appropriate for highly sensitive measurements of HTO, while keeping the total sample activity low. The noise equivalent concentration corresponds to a total activity 78 Bq inside the sample cell. The performance parameters are summarized in Table 1. The compact size and high sensitivity make the CEPAS setup also ideal for developing the method into a portable instrument. This would allow the opportunity for sensitive on-site measurements and monitoring of elevated HTO levels at nuclear sites.

## Declaration of Competing Interest

The authors declare that they have no known competing financial interests or personal relationships that could have appeared to influence the work reported in this paper.

## Data Availability

Data will be made available on request.

## Footnotes

1 A theoretical detection limit of 0.6 ppb was calculated for other HTO lines, but not demonstrated in the article.
